# Do privacy assurances work? a study of truthfulness in healthcare history data collection

**DOI:** 10.1371/journal.pone.0276442

**Published:** 2022-11-09

**Authors:** Tamara M. Masters, Mark Keith, Rachel Hess, Jeffrey L. Jenkins

**Affiliations:** 1 Department of Marketing, University of Utah, Salt Lake City, Utah, United States of America; 2 Department of Information Systems, Brigham Young University, Provo, Utah, United States of America; 3 Division of Health System Innovation and Research, University of Utah, Salt Lake City, Utah, United States of America; Taipei Medical University, TAIWAN

## Abstract

Patients often provide untruthful information about their health to avoid embarrassment, evade treatment, or prevent financial loss. Privacy disclosures (e.g. HIPAA) intended to dissuade privacy concerns may actually increase patient lying. We used new mouse tracking-based technology to detect lies through mouse movement (distance and time to response) and patient answer adjustment in an online controlled study of 611 potential patients, randomly assigned to one of six treatments. Treatments differed in the notices patients received *before* health information was requested, including notices about privacy, benefits of truthful disclosure, and risks of inaccurate disclosure. Increased time or distance of device mouse movement and greater adjustment of answers indicate less truthfulness. Mouse tracking revealed a significant overall effect (p<0.001) by treatment on the time to reach their final choice. The control took the least time indicating greater truthfulness and the privacy + risk group took the longest indicating least truthfulness. Privacy, risk, and benefit disclosure statements led to greater lying. These differences were moderated by gender. Mouse tracking results largely confirmed the answer adjustment lie detection method with an overall treatment effect (p < .0001) and gender differences (p < .0001) on truthfulness. Privacy notices led to decreased patient honesty. Privacy notices should perhaps be administered well before personal health disclosure is requested to minimize patient untruthfulness. Mouse tracking and answer adjustment appear to be health care lie-detection methods to enhance optimal diagnosis and treatment.

## Introduction

Consider these hypothetical scenarios: A patient indicates they are in pain from an old injury that requires narcotics; however, a controlled substance database search reveals he was given prescriptions for hydrocodone and fentanyl four days ago in another clinic. Another patient does not reveal using herbal remedies and is ordered an anticoagulant for a deep vein thrombosis. In both cases, the course of patient treatment might differ if the patients had been more truthful when answering the personal health questions or the clinicians used a method that alerted them to possible untruthful responses.

Accurate information is an important contributor to accurate diagnosis and treatment. Yet more than three quarters of patients withhold or lie about symptoms, intake, activities, and medication, which can have significant implications for their health care [1–4]. Research estimates that incorrect medications, possibly given as a result of false information provided, affect 1.5 million people, adding $3.5 billion in additional annual health care costs [2]. A wealth of studies have described the dilemma of patient lying but not provided a method to detect these lies [5].

Known causes of patient lying include a desire to avoid embarrassment, obtain or avoid treatment, or avoid financial costs [6]. To combat these lies, researchers have recommended that patient privacy be clearly signaled and assured [3, 6]. However, elaboration likelihood (ELM) theory [7] and related privacy information research [8–10] suggest that privacy assurances can have the exact opposite effect. Individuals typically analyze information through a *peripheral* route using mental shortcuts rather than logical reasoning. Objective information can cause people to shift their information processing to a *central* route with greater cognitive focus on the merits of the stimuli. Privacy notices can act as such a stimuli and cause consumers to withhold personal information and lie [11, 12]. In healthcare, privacy such as Health Insurance Portability and Accountability Act (HIPAA) notifications, may lead to greater misdiagnoses and treatment.

We conducted a randomized trial testing the provision of privacy notification on lying in healthcare communication. We hypothesized that health care notifications compared to no notifications would lead to more lying.

## Materials and methods

### Study design methodology, context, and measures

We recruited participants from the Amazon Mechanical Turk (MTurk) platform, as these are shown to be reliable online panels [13–15]. Participants were randomly assigned to receive one of six stimuli: control (no privacy, risk or benefit statement), benefit (statement about the benefits of accurate information disclosure), risk (statement about the health risks of inaccurate information disclosure), privacy (a traditional privacy notification with seal image), privacy + benefit, and privacy + risk. For example, a question about weight with a benefit statement read: “What is your weight? *Accurately answering this will increase the likelihood of a correct diagnosis*.” A risk statement read: “What is your weight? *Inaccurately answering this will increase the likelihood of an incorrect diagnosis*.” A privacy statement read: “What is your weight? *We will not share or sell this personal health information with anyone*. *We will comply with all HIPAA regulations regarding the protection of your data*.” (see S2 Appendix).

These additional stimuli concerning the risks and benefits of (in)accurate disclosure are practically motivated by privacy research showing that telling consumers *why* information is needed will have a significant effect on disclosure accuracy [8, 10]. The distinction between benefit and risk framing, coming from behavioral economics (i.e., prospect theory [16]), explains that people overestimate benefits and underestimate risks, thus providing a relevant subhypothesis to our study.

After agreeing to an IRB–approved modified consent form (designed to hide the true purpose of the study to detect lying), participants were asked to complete a depression survey, the CESD-10 depression scale [17]. This depression scale was used in order to simulate the pretense of the experiment. This context was chosen because depression is relevant to a large percentage of the world’s population [18]. Similarly, online patient portals and electronic health information requests are becoming the norm [19]. The participants were asked to disclose eight items of personal health information of varying levels of sensitivity, including their weight, height, alcohol intake, illegal drug use, prescription drug abuse, smoking, exercise, and sexual activity in the context of the stimulus statement (see S1 Appendix).

### Measures

To determine participant honesty, we tracked distance and speed of mouse movements while answering each question. Mouse tracking is an accurate indicator of cognitive and emotional stress [20] and deceit [21] while disclosing personal information. The brain shares its ongoing work with the motor cortex, and hand movement is a predictor of cognitive processing, decision conflict, and deception [21, 22]. Lying requires greater cognitive processing than providing truthful responses, as individuals evaluate the truth and select a lie. This leads to cognitive decision conflict that can be detected on a laptop or tablet device that has a mouse or touchpad. Biomechanical mouse movement has comparable efficacy to measuring electrodermal activity (e.g. polygraphs) for identifying deception [20].

Mouse tracking hand movements include both trajectory (distance) and time for a response. Participants’ procedural memory leads them to begin with an accurate response, but then alters the trajectory to an inaccurate response. Thus, lying is indicated by greater time and less direct movement (greater distance). These metrics are collected using JavaScript code, which detects all mouse movements at a millisecond precision rate [20, 21]. This software is hidden to participants as they answered the personal health history indicators (height, weight, alcohol, illegal drug use, prescription abuse, smoking, exercise, and sex). Mouse movement distance and total time to response were normalized to z-scores separately for each participant across all survey questions. Thus, a positive score indicates the participant took longer answering that question than their average of all other survey questions. This participant-level normalization is important because people differ considerably in how they interact with computers, and hand movement precision. Importantly, this tracking is completely undetectable by the participant with no delay or lag. Although mouse tracking is a proven technique for collecting objective measures of truthfulness [21], we complemented the mouse tracking measures with an answer adjustment method—similar to those used in consumer information privacy experiments [23]—that involves directly asking participants whether they were truthful. After participants made their initial disclosure decisions, they were given a summary of their answers in a read-only format and asked to indicate how over- or understated each initial response was (e.g., “You indicated that your current weight is 185 lbs. How overstated or understated is that value?”) on a scale from -5 (understated) to 5 (overstated). The absolute value of their response represents the extent to which participants’ initial response deviated from the truth. These responses help to validate the mouse tracking results and provide a more holistic measure of truthfulness.

### Analysis

The trial was designed to provide at least 80% power in analysis of the effect of treatments. Based on a 3x2 factorial design, 42 individuals are needed per group (252 total) to provide (80%) power to detect a 25% effect size or difference in our experimental variables at a significance of (.05). Additional samples were collected because of the potential risk of technical issues collecting mouse tracking data.

The dependent variable—patient truthfulness was measured using two methods: biometric mouse movement [20–22, 24] and a stated answer adjustment method [23]. Biometric mouse movement was measured using two factors: distance the mouse moved and the time it took to arrive at the final response. A person not reporting the truth will take more time and greater distance as they move the mouse on an initial trajectory to the truth and correct themselves to go to the non-truthful answer they decide to share. Z scores, calculated for each response on the survey, were analyzed using ANOVA and Tukey’s post hoc test which compares each mean to every other mean [20]. The stated answer adjustment metric of how much the individual self corrects an initial false response was analyzed with ANOVA across means and MANOVA for gender differences across each condition.

## Results

### Lie detection through mouse movement

**Fig 1:**
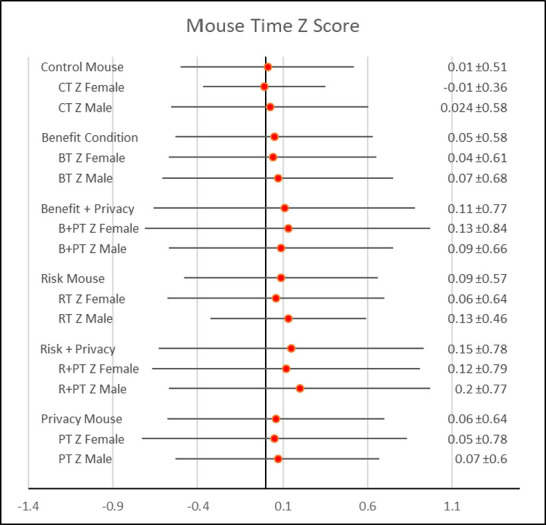
Forest plot of mouse movement time z scores by treatment and gender.

Six hundred eleven participants completed the survey (M_age_ = 36 years, range: 18–78 years; 64% female). Mouse tracking data were fully captured on 504 participants (see Table 1). The other 107 participants had minor technical issues from either their device or poor network connectivity. This is not unexpected as devices and networks vary greatly in terms of the browser settings, firewall and malware which can conflict with the JavaScript mouse tracking software. Answer adjustment was successfully measured in 587 participants as 24 did not fully complete the survey. Fig 1 illustrates the significant difference (p = .001) in average time z score to response for the eight personal health history questions grouped by stimuli treatment.

**Table 1:** Count of individuals randomly assigned to each condition.

No statements	Benefit statement	Cost statement	Privacy statement	Benefit + Privacy	Cost + Privacy	Total
105	97	104	105	101	99	**611**
17.2%	15.9%	17%	17.1%	16.5%	16.2%	

Comparing treatments, the *control* treatment (no stimuli) had the lowest mouse time to response ($\bar{\text{x}}$ = 0.01), followed by the *benefit* stimulus ($\bar{\text{x}}$ = 0.05), *privacy* (similar to a short HIPAA reminder, $\bar{\text{x}}$ = 0.06), and *negative effect (i*.*e*., *risk) of inaccurate disclosure* ($\bar{\text{x}}$ = 0.09). Privacy, risk, and the combination of privacy and benefit ($\bar{\text{x}}$ = 0.11) and privacy and risk ($\bar{\text{x}}$ = 0.16) led to greater time spent answering the questions than the control. These increases were significant (F = 4.64, p < .001) and indicate lack of truthfulness after statements of privacy, risk and benefit plus risk. Fig 1 shows the results by treatment and gender.

Mouse movement distance revealed similar results. Although there was no significant difference in z scores across all treatments (p = .19), compared to the control treatment, benefit + privacy (p = .08) and the risk + privacy (p = .06) led to significantly greater mouse movement, indicating lack of truthfulness. Results are seen in Table 2 and Fig 1.

**Table 2:** Mouse tracking time and distance to final response (z score).

Treatment	Total Time z score	difference from control	Total Distance z	difference from control
Control	0.01 (SD .51)		0.07 (SD .56)	
Benefit	0.05 (SD .58)	p = .26	0.11 (SD .55)	p = .28
Benefit + Privacy	0.11 (SD .77)	p = .001	0.14 (SD .66)	p = .08
Risk	0.09 (SD .57)	p < .0001	0.12 (SD .52)	p = .13
Risk + Privacy	0.15 (SD .79)	p < .0001	0.14 (SD .63)	p = .06
Privacy	0.06 (SD .64)	p = .04	0.10 (SD .57)	p = .23

**Fig 2:**
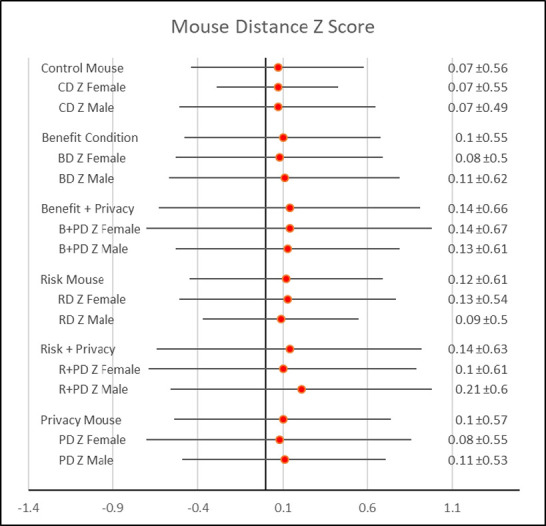
Forest plot of mouse movement distance z score by treatment and gender.

Gender had a significant effect on both time to response (F = 3.86, p = .048) and mouse distance traveled (F = 9.35, p = .002). In particular, men exhibited greatest mouse distance (${\bar{\text{x}}}_{\text{men}}$ = .21 > ${\bar{\text{x}}}_{\text{women}}$ = .07) and time (${\bar{\text{x}}}_{\text{men}}$ = .20 > ${\bar{\text{x}}}_{\text{women}}$ = .13) to response across treatments than women. Fig 2 illustrates differences by each treatment. In particular, men had greater mouse movements in the benefit, privacy, and risk + privacy treatments, while women had greater mouse movements in the risk treatment.

As Fig 2 shows, the total time spent answering the questions almost mirrors the mouse movement distance results, except that men moved their mouse less during the risk and benefit + privacy treatment.

### Lie detection through answer adjustment

**Fig 3:**
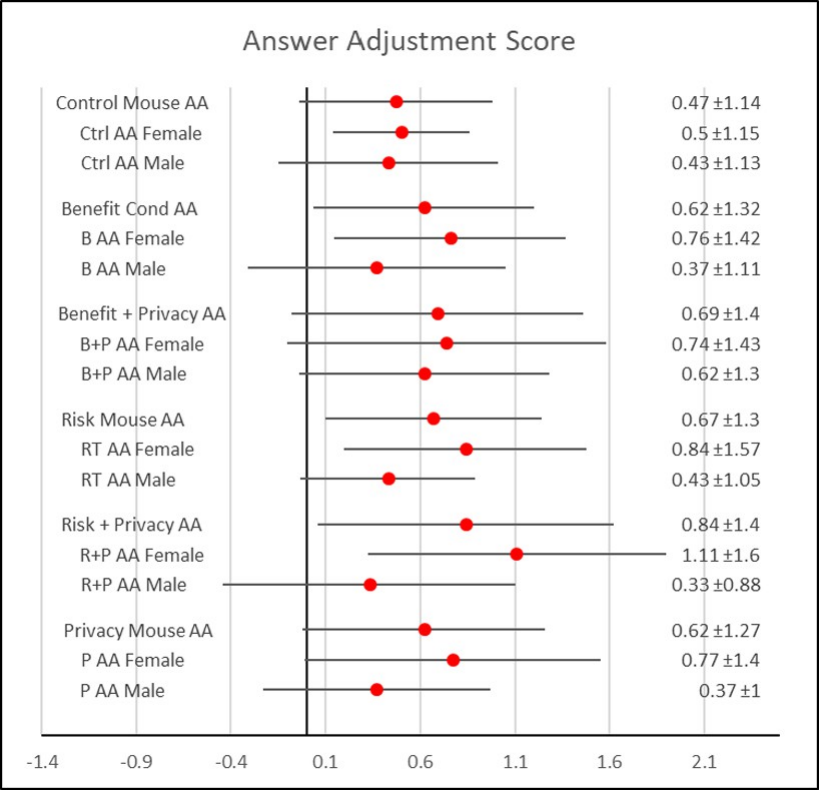
Forest plot of answer adjustment by treatment and gender.

Generally, the stated answer adjustment results are consistent with the mouse tracking findings with the exception of the gender effects. There was a significant overall main effect of treatment (F = 5.67, p < .0001). The control treatment set a base level of lying at $\bar{\text{x}}$ = 0.47. Similar to the mouse tracking data, the greatest adjustment occurred between the control versus risk + privacy treatment (${\bar{\text{x}}}_{\text{risk}+\text{privacy}}$ = .84 > $\bar{\text{x}}$_control =_ .47; F = 27.64, p < .0001). All other comparisons to the control were statistically significant and followed similar patterns as the mouse tracking data. Results are seen in Table 3 and Fig 3.

**Table 3:** Answer adjustment measures (abs) by treatment for combined questions: Weight, EtOH, drugs, OD, smoke, exercise, sex.

Treatment	AA (Abs)	difference from control
Control	0.47 (SD 1.17)	
Benefit	0.62 (SD 1.32)	p = .01
Benefit + Privacy	0.69 (SD .95)	p = .006
Risk	0.67 (SD 1.3)	p = .009
Risk + Privacy	0.84 (SD 1.40)	p = .000
Privacy	0.62 (SD 1.27)	p = .008

Gender had a significant main effect (F = 75.66, p < .0001) on answer adjustment. However, in contrast with the mouse data, men adjusted less than women (${\bar{\text{x}}}_{\text{men}}$ = .42 < ${\bar{\text{x}}}_{\text{women}}$ = .79; F = 75.66, p < .0001). Fig 3 depicts the gender differences by treatment. Men claimed to lie less than women in their initial responses in every treatment with the greatest difference in the risk + privacy stimuli.

## Discussion

Overall, mouse movement results indicate that each stimuli—privacy notifications and benefit/risk statements—led to less honesty in personal health information disclosure. Both mouse speed and distance results indicate that stimuli such as privacy notices, and statements regarding the risks/benefits of (un)truthful personal health information disclosure lead to greater decision conflict and deception in response to personal health questions. Stated answer adjustment confirms that this decision conflict represented by the mouse tracking data is, indeed, actual lying about personal health activity. This lying can lead to a greater number of misdiagnoses and poor outcomes [1, 25]. Furthermore, we find that messages that are intended to calm and assure patients of their privacy rights may arguably be the strongest stimuli leading to patient lying.

In support of prior research which shows that men and women both lie, but in different ways [26, 27], the results of these two measures of truthfulness tell opposing stories: (1) men lie more than women when measured by mouse movement, and (2) women lie more than men when measured by answer adjustment—which requires some degree of subjective honesty compared to the more objective measures. We offer multiple explanations about why the mouse tracking and answer adjustment gender differences may be skewed. One reason may be that core gender differences exist in how the human-computer interface of a mouse affects information [28]. In other words, perhaps women simply take less time using a mouse than men in general. Thus, they might still be exhibiting high decision conflict but are simply more adept at using a mouse or spend less time making decisions in general. If so, this would suggest that women face greater decision conflict and lie more than men, when juxtaposed to the answer adjustment data [29]. This is a potential topic for future research.

Women are known to have greater desire to be viewed favorably compared to men [30]. This social desirability bias is demonstrated when participants respond in ways they think are favorable to the administrator [30]. Therefore, women would be more likely to answer the answer adjustment questions accurately. If true, then the mouse tracking results are correct and men simply do not want to admit they’re lying when faced with the opportunity to correct their initial answers. This interpretation is supported by research of online profiles where men are known to be less truthful than women [31].

Regardless, under the assumption that men and women lie equally, risk + privacy information leads to the most adjustment from their initial response to one they determine is more truthful by women and benefit + privacy information to the most adjustment by men. Under the assumption that men’s and women’s propensity to lie differs, risk + privacy information leads to the least adjustment by men, and the control condition (no mention of benefit, risk, or privacy) yields the least adjustment by women. Furthermore, the results reveal that asking for accuracy adjustment may trigger more truthfulness from women than men.

Both regulators and clinicians might want to take these findings into consideration. In the long run, regulators should reconsider the requirements about disclosing HIPAA information. In the short run, this research suggests that perhaps clinicians should not have patients read and sign privacy policies (HIPAA) at the time of data collection. Having patients sign HIPAA notices immediately before talking with the clinician or discussing privacy during the visit may lead to increased lying, which in turn may result in misdiagnoses or complications from inappropriate treatment.

Finally, this study also illustrates how lie detection methods such as mouse tracking are useful when considering patient health disclosures. When clinicians review patient information, systems developed to detect lying could flag data points that may have been disclosed when over thinking due to decision conflict and indicate that lying may have occurred.

This study has a number of limitations. First, this is an experimental setting and solely online, but this method has proven valid with electrodermal activity used for polygraph examinations [20]. Second, this was only run in a single medical context of health history use for mental health. Future studies could use this method in varied clinical settings to validate our findings and investigate if there are differences with clinician fact-to-face lie detection. In addition, while there were significant differences in time to response and answer adjustment, results were mixed with non-significant distance to results measured. Further use of these tools may help health care workers understand when patients are less than truthful.

## Conclusions

This research reveals the negative impact of benefit, risk, and privacy statements on truthfulness and show gender differences on truthfully answering health history questions. It also illustrates ways to detect deception and increase patient truthfulness to ensure optimal diagnosis and treatment. These results and tools could be considered when collecting data from patients.

## Supporting information

S1 Appendix Health assessment questions. (TIF)

S2 Appendix Statements for treatments. (TIF)
