# Supplementary figures and images for: Do privacy assurances work? a study of truthfulness in healthcare history data collection
Source: PLoS One. 2022 Nov 9;17(11):e0276442. doi: 10.1371/journal.pone.0276442 (PMC9645639; doi:10.1371/journal.pone.0276442)

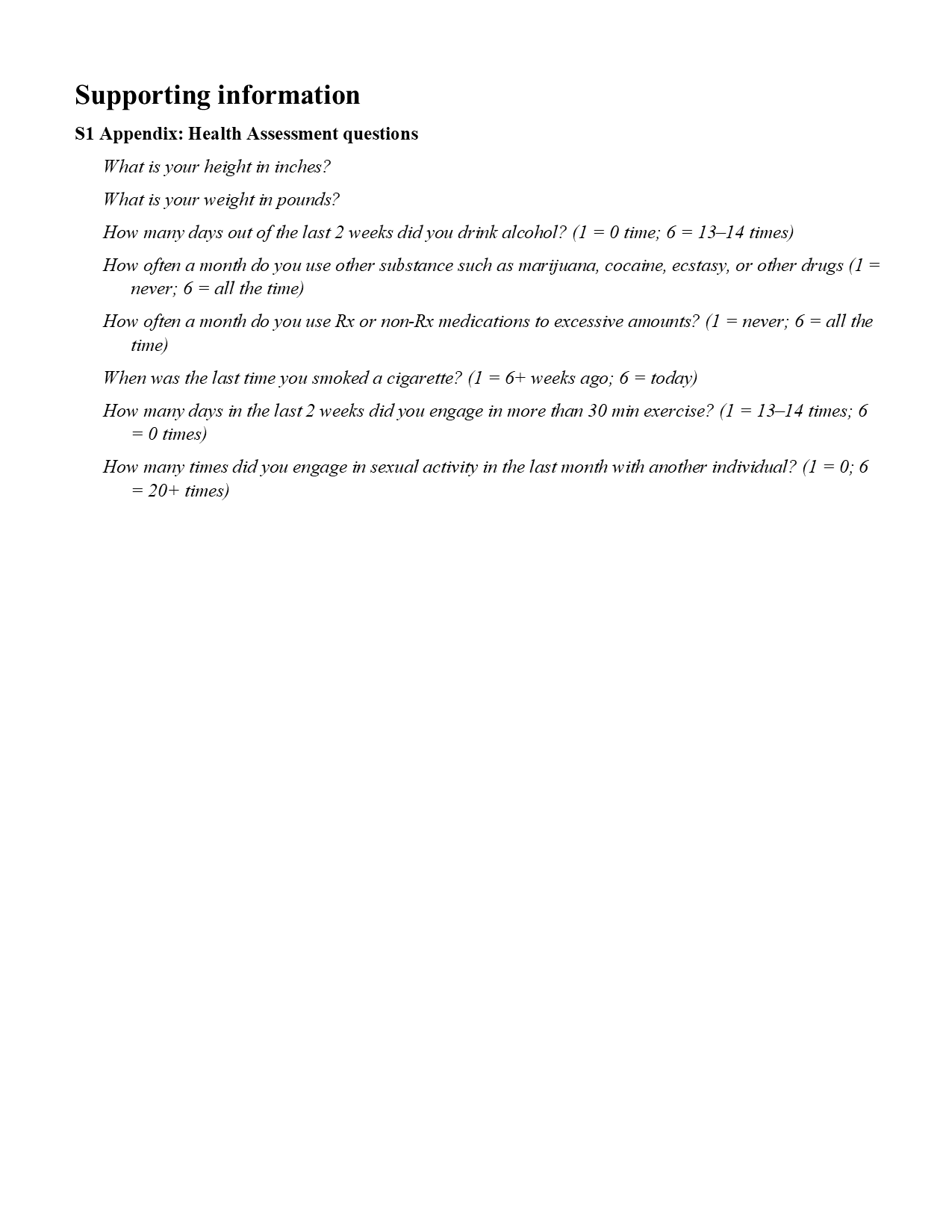

Supplement: S1 Appendix — (TIF) [file pone.0276442.s001.tif]

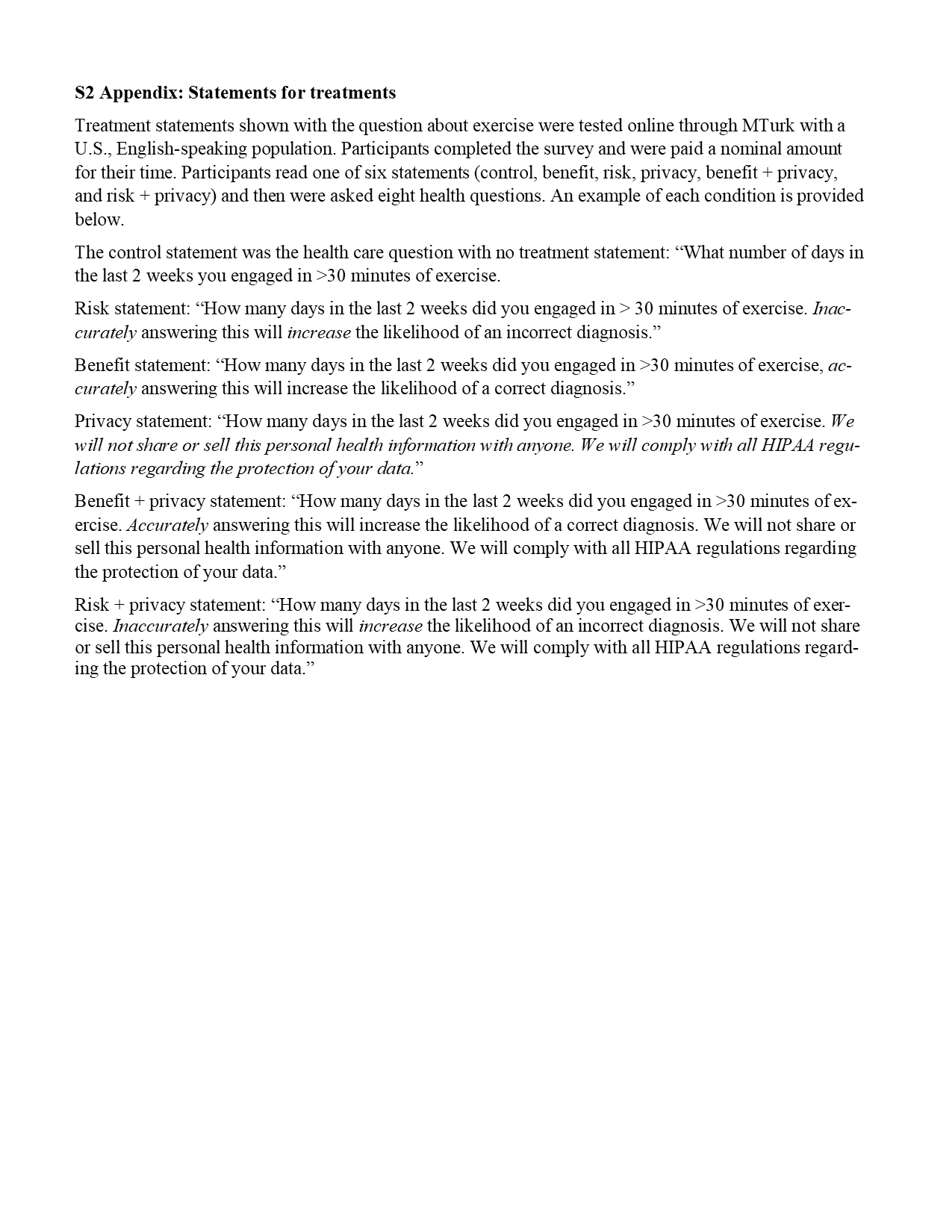

Supplement: S2 Appendix — (TIF) [file pone.0276442.s002.tif]
